# Centromeric AA motif in KIR as an optimal surrogate marker for precision definition of alloimmune reproductive failure

**DOI:** 10.1038/s41598-024-53766-x

**Published:** 2024-02-09

**Authors:** Raquel Gil Laborda, Edgard Rodríguez de Frías, Nabil Subhi-Issa, Elena Carrillo de Albornoz, Elena Meliá, Marcos Órdenes, Victoria Verdú, Juan Vidal, Esther Suárez, Isabel Santillán, Daniel Ordóñez, David Pintado-Vera, Victoria González Villafáñez, Ángel Lorenzo, Manuel Fariñas, Mario Rodríguez-Paíno, María Núñez Beltrán, Áurea García Segovia, Ainhoa del Olmo, Fernando Martín Cañadas, Rosa Daurelio, Alfonso de la Fuente, José Manuel González Casbas, Vega Cabezuelo, Francisco Ros Berruezo, Miguel Ángel Moreno Hidalgo, Silvia Iniesta, Beatriz Bueno, Álvaro Martínez Acera, Alexandra Izquierdo, José Luis Vicario, Miguel Fernández-Arquero, Silvia Sánchez-Ramón

**Affiliations:** 1 Department of Immunology, IML and IdISSC, Hospital Clínico San Carlos, Madrid, Spain; 2 Clinical Immunology Unit, Hospital Ruber Internacional, Madrid, Spain; 3 Woman Unit, Hospital Ruber Internacional, Madrid, Spain; 4 Assisted Reproductive Unit, Hospital Ruber Internacional, Madrid, Spain; 5 Clínica GINEFIV, Madrid, Spain; 6 Clínica IVF, Madrid, Spain; 7 Instituto Europeo de Fertilidad (IEF), Madrid, Spain; 8 Sterility and Infertility Unit, Hospital Quiron Salud, Pamplona, Spain; 9 Clínica Hemomadrid, Madrid, Spain; 10 Sanitas Assisted Reproduction Unit, Clínica Sanitas Millenium Alcobendas, Madrid, Spain; 11 Instituto Malavé de Ginecología, Málaga, Andalucía Spain; 12 Assisted Reproduction Unit in HM Fertility Center, Gabinete Velázquez, Madrid, Spain; 13 Centro de Trasfusión de la Comunidad de Madrid, Madrid, Spain

**Keywords:** Immunoglobulin-Like Receptor, Killer Cell, Natural Killer Cells, HLA-C Lymphocyte Antigen, Unexplained Recurrent Pregnancy Loss, Recurrent Pregnancy Loss, Recurrent Implantation Failure, Diagnosis, Disease prevention, Genetics, Immunology

## Abstract

Throughout pregnancy, the decidua is predominantly populated by NK lymphocytes expressing Killer immunoglobulin-like receptors (KIR) that recognize human leukocyte antigen-C (HLA-C) ligands from trophoblast cells. This study aims to investigate the association of KIR-HLA-C phenotypes in couples facing infertility, particularly recurrent pregnancy loss (RPL) and recurrent implantation failure (RIF), in comparison to a reference population and fertile controls. This observational, non-interventional retrospective case–control study included patients consecutively referred to our Reproductive Immunology Unit from 2015 to 2019. We analyzed the frequencies of KIR and HLA-C genes. As control groups, we analyzed a reference Spanish population for KIR analysis and 29 fertile controls and their male partners for KIR and HLA-C combinations. We studied 397 consecutively referred women with infertility and their male partners. Among women with unexplained RPL (133 women) and RIF (176 women), the centromeric (cen)AA KIR genotype was significantly more prevalent compared to the reference Spanish population (*p* = 0.001 and 0.02, respectively). Furthermore, cenAA was associated with a 1.51-fold risk of RPL and a 1.2-fold risk of RIF. Conversely, the presence of BB KIR showed a lower risk of reproductive failure compared to non-BB KIR (OR: 0.12, *p* < 0.001). Women and their partners with HLA-C1C1/C1C1 were significantly less common in the RPL-Group (*p* < 0.001) and RIF-Group (*p* = 0.002) compared to the control group. Moreover, the combination of cenAA/C1C1 in women with C1C1 partners was significantly higher in the control group than in the RPL (*p* = 0.009) and RIF (*p* = 0.04) groups, associated with a 5-fold increase in successful pregnancy outcomes. In our cohort, the cenAA KIR haplotype proved to be a more accurate biomarker than the classic AA KIR haplotype for assessing the risk of RPL and RIF, and might be particularly useful to identify women at increased risk among the heterogeneous KIR AB or Bx population. The classification of centromeric KIR haplotypes outperforms classical KIR haplotypes, making it a better indicator of potential maternal–fetal KIR-HLA-C mismatch in patients.

## Introduction

Recurrent Pregnancy Loss (RPL) and Recurrent Implantation Failure (RIF) are discrete clinical conditions characterized by distinct gynecological and immunological risk factors^1^. RPL is a multifaceted reproductive disorder with diverse potential causes and contributing factors that extend beyond embryonic abnormalities. Nevertheless, the etiology remains unexplained in nearly 50% of cases^2^. Conversely, RIF encompasses various risk factors, including maternal factors such as age, BMI, stress, and tobacco use, along with embryonic genetic abnormalities, which contribute to 50–70% of implantation failures^3,4^. Despite the advances in in vitro fertilization (IVF) and preimplantation genetic testing (PGT), more than 30% of euploid embryos fail to result in a live birth. This suggests the involvement of immunological, endocrine, or hematologic factors, such as obstetrical antiphospholipid syndrome (OAPS) and thyroid disorders. In the past decade, various maternal inflammatory processes have been associated with RPL and RIF, encompassing a proinflammatory profile of peripheral natural killer (NK) cells, T lymphocyte differentiation, and altered function of uterine NK (uNK) cells^5,6^.

The first trimester of pregnancy witnesses a significant presence of uNK cells in the endometrium, constituting about 70% of maternal immune cells during the implantation window^7–9^. These cells play a pivotal role in placental development and the remodeling of uterine spiral arteries in early pregnancy. Disrupted uNK cell function can lead to defective uterine spiral artery remodeling and shallow placental implantation, which are associated with serious obstetric syndromes beyond RPL, like preeclampsia (PE)^10^, fetal growth restriction (FGR), and stillbirth (S)^7,11^. More recently, uterine innate lymphoid cells (uILCs) including subsets of tissue-resident uNKs cells and non-NK ILCs, mainly ILC1, ILC3 and LTi-like cells, interact with ligands expressed on fetal extravillous trophoblasts (EVT)^12^.

Human Leukocyte Antigens (HLA) expressed by the EVT in early pregnancy, such as oligomorphic HLA-E and HLA-G^13^, alongside the highly polymorphic HLA-C, play a pivotal role in the selective recognition by uNK cells. Killer Immunoglobulin-like Receptors (KIRs), a distinctive set of receptors on uNK cells, engage with HLA-C at the maternal–fetal interface. Featuring both activating and inhibitory motifs, KIR finely modulate uNK cell activity and contribute to early placental development^5^. KIRs interact with various HLA-C allotypes, categorized into C1 and C2 groups^14,15^. While HLA-C1 molecules have minimal impact on uNK cell activity, the binding of HLA-C2 to KIR2DL1 or KIR2DS1 strongly inhibits or activates uNK cells, respectively^16^.

**Figure 1:**
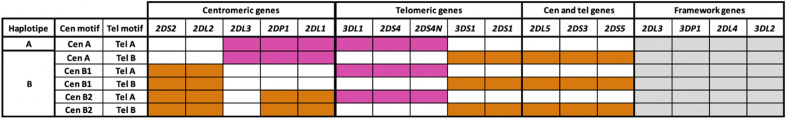
The KIR gene cluster is characterized by its centromeric (Cen) and telomeric (Tel) gene content motifs. Within the KIR locus, the genes are organized in a distinct manner. The centromeric and telomeric regions are separated by a unique recombination site sequence, which allows for the exchange of genes between the centromeric and telomeric motifs. The common motifs contain a set of conserved framework genes, shaded in gray, while the B haplotype genes are represented in orange, and the A haplotype genes are shown in pink. The centromeric genes crucial for haplotype generation, namely 2DL2, 2DL3, and 2DS2, play a pivotal role. The co-occurrence of 2DL2 and 2DL3 results in the AB haplotype, while the presence of solely 2DL3 yields the AA haplotype, and solely 2DL2 leads to the BB haplotype. The 2DS2 allele exclusively manifests within the AB or BB haplotypes. Turning to the telomeric haplotype, key genes involved in its determination include 3DL1, 3DS1, 2DS4, and 2DS1. Specifically, the AB haplotype emerges when a concurrent presence of 3DL1 and 2DS4 accompanies 3DS1 and 2DS1. Conversely, the exclusive presence of 3DL1 and 2DS4 delineates the AA haplotype, while the co-presence of 3DS1 and 2DS1 characterizes the BB haplotype.

KIR genes are inherited in linkage disequilibrium, forming two haplotypes: haplotype A, distinguished by a fixed combination of nine genes (7 protein-coding genes and 2 pseudogenes) predominantly exerting inhibitory effects, including KIR2DL1; and haplotype B, encompassing a more diverse KIR gene content, exerting non-effect or activator effects on NK cells (Fig. 1)^17^. Further subdivision of each KIR haplotype into centromeric and telomeric motifs contributes to genetic polymorphism^18,19^. The recombination of these motifs accounts for most KIR genotypes observed in the European population. KIR2DL1 alleles inherited within the centromeric motifs of haplotype A (cenA) exert stronger inhibitory and educating effects on uNK cells compared to those inherited within centromeric motifs of haplotype B (cenB). This distinction arises from the specific combination of receptors defining each haplotype^16,20,21^.

Previous research observed an increased frequency of KIR AA in women experiencing RPL^22^, as well as in cases of PE^10,23,24^. Conversely, other studies reported a decreased frequency of inhibitory KIRs^25,26^ or a prevalence of activating KIRs in patients with RPL^27^. Recent findings also indicated a higher miscarriage rate in women with the KIR AA haplotype undergoing IVF, although KIR frequency in the RIF group was not reported^28^. In a study by Morin et al., the KIR AA and HLA-C2 combination strongly correlated with subsequent embryo loss in a retrospective cohort of nearly 700 individual euploid embryo transfers, while KIR Bx (comprising AB and BB) was linked to 30% of pregnancy loss independently of HLA-C^29^.

While the negative impact of the KIR AA haplotype on reproduction is well documented, the influence of other KIR gene combinations remains less explored. This knowledge gap can be attributed, in part, to varying classifications of KIR genotypes. According to the World Health Organization (WHO) and the Human Genome Organization Subcommittee on KIR Nomenclature, haplotype A is defined by a fixed combination of nine genes, whereas haplotype Bx encompassing any other KIR gene combination, is less distinctly characterized and displays considerable heterogeneity^30^. Recent research has made significant strides in comprehending the structure and variability patterns of the KIR gene complex, culminating in an enhanced classification system based on centromeric and telomeric motifs (Fig. 1).

In this study, we sought to assess the prevalence of two cenAA in a substantial cohort of women experiencing unexplained RPL and RIF. Our findings suggest that cenAA could potentially serve as a more robust biomarker for predicting adverse pregnancy outcomes compared to the classical AA haplotype. Importantly, this may be especially valuable in identifying women at an elevated risk within the heterogeneous KIR AB or Bx population.

## Materials and methods

### Study design

Our study was designed as a retrospective, observational case–control study, specifically aimed at assessing whether the classification of KIR into centromeric and telomeric motifs introduces additional clinical significance beyond the classical KIR classification.

### Subjects

Our study group included women with RPL and RIF, S and FD of unknown etiology along with their partners. These individuals were consecutively referred for immunological evaluation at a single Reproductive Immunology Unit of Hospital Ruber Internacional between 2015 and 2019. Routine clinical assessments were conducted on these patients, and the findings were systematically documented for subsequent group analyses. The reference population group for KIR genotype was derived from a study by Vilches et al.^31^, focusing on a Spanish Population. For KIR-HLA-C combinations comparative purposes, our control group consisted of 29 women and their partners with a history of at least two natural uncomplicated pregnancies resulting in the birth of at least two healthy newborns. This group was used for further analysis and comparisons from our laboratory^32^.

The study adhered to the Declaration of Helsinki. For the study group, the Institutional Ethics Committee of Clinical Research of the Center (CEIC del Hospital RUBER Internacional) approved the study protocol (Project No. Sa-16290 / 19-EC: 401, code: MARINMFGR_01_2020). Informed consent was waived by the center's ethics committee (CEIC del Hospital RUBER Internacional) due to the anonymized nature of the database and the impracticality of contacting each patient to obtain specific consent for the retrospective research. As for the healthy control group, we examined fertile women and their partners in the setting of another project (FIS PI19/01450). The study protocol including fertile healthy controls was approved by the Ethics Committee of Hospital Clínico San Carlos, and all subjects provided signed informed consent.

In this context, we defined the following groups: Sterility (S): Defined as the inability to achieve a clinical pregnancy after 12 months of regular, unprotected sexual intercourse, following the International Glossary on Infertility and Fertility Care^33^. Recurrent Pregnancy Loss (RPL): Defined as the loss of two or more pregnancies, including non-visualized pregnancy losses, according to the European Society for Human Reproduction and Embryology (ESHRE) Guidelines. Recurrent Implantation Failure (RIF): Defined as the failure to achieve a clinical pregnancy after more than 3 high-quality embryo transfers or after the transfer of ≥ 10 embryos in multiple transfers in women below 40 years of age. Fetal Death (FD): Defined as a composite outcome that included women with a history of late fetal loss (between 22 and 28 weeks of pregnancy) and stillbirth (after 28 weeks of gestational age)^34^.

### HLA-C and KIR genotyping

The KIR and HLA-C genotypes of both woman and their male partner of each group were determined. DNA isolation was performed with the MagNA Pure appliance, an automated system that allows DNA isolation from peripheral blood. For the isolation, we used the Isolation Kit I -Large Volume (Ref: 03730972001 Roche Diagnostics GmbH).

Before genotyping HLA-C and KIR, a PCR-SSP is performed from the following specific Kits, Lifecodes HLA-C eRES SSO Typing Kit (Ref: 628921, Immucor Medizinische diagnostic Gmbh), and Lifecodes KIR Typing Kit (Ref: 545110R, Immucor Medizinische diagnostik Gmbh).The HLA-C and KIR typing were performed using the Luminex equipment; the testing for patients was carried out at the Centro de Transfusiones de la Comunidad de Madrid, while the controls were tested at the Hospital Universitario Clínico San Carlos, Madrid. The DNA samples were analyzed for the presence of the 14 KIR genes (2DL1, 2DL2, 2DL3, 2DL4, 2DL5, 2DS1, 2DS2, 2DS3, 2DS4, 2DS5, 3DL1, 3DL2, 3DL3, 3DS1) and 2 pseudo-genes (2DP1 and 3DP1).

To ensure the accuracy of the analysis, HLA-C alleles were categorized into two groups based on the amino acid present at position 80 of the alpha domain of the molecule. Those containing asparagine were assigned to the C1 group, while alleles containing lysine were assigned to the C2 group.

Individual KIR gene analysis was conducted, and the combinations of genes were compared to haplotypes previously identified in the South European population. As part of the quality control process, KIR genotype results with missing framework KIR genes were excluded from the analysis.

As shown in Fig. 1, the assignment of cenA or cenB motifs relied on the identification of KIR2DL3 or KIR2DS2-2DL2, respectively. Similarly, in the telomeric region, the assignment of telA or telB motifs was determined by the identification of KIR3DL1-2DS4 or KIR3DS1-2DS1, respectively. Genes such as KIR-2DS3, KIR-2DS5, and KIR-2DL5 were considered components of cenB and telB motifs, irrespective of their specific positions among adjacent KIR genes. Patients with two cenA and two telA motifs were categorized as having the AA genotype, while those with two telB and two telB motifs were classified as having the KIR BB genotype. The AB (Bx) genotype was assigned when any other combination of A and B motifs was present. Additionally, the gene dose of KIR 2DL1A was estimated based on the number of cenA motifs identified in each patient, following the method previously described by Moffet^35^.

### Statistical analysis

The analysis conducted in this study primarily focused on the computation of frequencies associated with various KIR genotypes, as well as centromeric and telomeric motifs. We studied 397 patients with uRRF consecutively referred to our Reproductive Immunology Unit between 2015 and 2019 and compared the KIR genotype against a reference population. We calculated the statistical power of cenAA for this cohort of patients with respect to reference Spanish population, resulting in a statistical power of 90% with a confidence interval of 95%. These frequencies were determined within the study cohort, comprising individuals with RPL and RIF, and specific subgroups within this cohort. Subsequently, a comparative assessment was made by juxtaposing these frequencies against those documented for the Spanish population and a fertile cohort of women and their couples. Demographic, clinical, and immunological data were subjected to comparison using the chi-square (χ^2^) test, one-way analysis of variance (ANOVA), or the Kruskal–Wallis test, as deemed appropriate. Logistic regression was employed to scrutinize the correlation between KIR/HLA-C haplotypes and pregnancy outcomes, with odds ratios (OR) and 95% confidence intervals (CI) calculated. Stratified analysis was implemented to mitigate confounding factors. All statistical analyses were two-sided, with a significance level set at *P* < 0.05, indicating statistical significance. To address the issue of multiple comparisons, a Bonferroni correction was applied, adjusting the P-value to 0.0125 (0.05/4). The statistical analyses were executed utilizing SPSS (version 22.0; SPSS Institute, Chicago, IL, USA) and Stata (version 14.0; STATA Corp, College Station, TX, USA).

## Results

### Epidemiological and obstetrical features of the study subjects

In this investigation, we have systematically analyzed 397 patients with uRRF who were consecutively referred to our Reproductive Immunology Unit between 2015 and 2019. By comparing the KIR genotype of this cohort against a reference population, we aim to elucidate potential correlations and clinical implications associated with the specific centromeric and telomeric motifs. Their obstetrical history at the time of immunological evaluation revealed different diagnoses: 15.11% were diagnosed with primary sterility (S-Group); 33.50% with RIF (RIF-Group); 44.33% with RPL (RPL-Group); and 7.05% had a previous FD (FD-Group). Table 1 shows that all groups shared similar characteristics, allowing for comparability in further analysis.

**Table 1:** Clinical and demographical features of the study groups.

	S-group Nº = 60	RPL-group No = 133	RIF-group No = 176	FD-group No = 28	HC No = 29
Age (years)	36.7 ± 3.8	36.7 ± 3.8	35.8 ± 4.5	36.3 ± 4.5	37.9 ± 6.4
No. of miscarriages	3.0 ± 1.3	3.0 ± 1.3	0.3 ± 0.5		0
No. of IVF cycles	1.2 ± 2.0	1.2 ± 2.0	3.0 ± 1.4		0
No. of embryos	2.2 ± 1.4	2.2 ± 1.4	9.0 ± 4.2		NA

Values are expressed as mean ± SD.

*FD* fetal death, *HC* healthy controls, *IVF* in vitro fertilization, *NS* no significant, *RPL* recurrent pregnancy loss, *RIF* recurrent implantation failure.

We then narrowed our focus to patients without OAPS as the primary cause of recurrent infertility, aiming to minimize confounding factors associated with maternofetal HLA-C-KIR mismatch. Among the 321 women without OAPS, the distribution among diagnostic groups remained consistent: 14.95% diagnosed with S, 34.58% with FIR, 43.61% with RPL, and 6.85% with FD. Additional insight revealed that individuals with sterility pursued pregnancy at an average of 37.7 years, those with RIF had a mean of 2.2 previous good embryo transfers, and individuals with RPL experienced an average of 3 previous miscarriages (Table 1).

### Classic KIR genotypes in unexplained reproductive failure

Transitioning to the investigation of KIR genotypes, we computed frequencies for various KIR genotypes and compared them to reported frequencies in the Spanish population (Table 2). In the entire cohort of patients with unexplained reproductive failure (uRF), the KIR AB genotype predominated, observed in 63.22% (251/397) of cases, followed by KIR AA in 35.01% (139/397) and KIR BB in 1.76% (7/397) of women.

**Table 2:** Classic KIR haplotypes frequencies in each of the main study group and in the reference population.

KIR	Classic KIR haplotypes frequencies in comparison with reference population													
		Ref. P (n = 402)	uRF-group (n = 397)				RIF-group (n = 133)				RPL-group (n = 176)			
		N (%)	N (%)	*P*	OR	95%CI	N (%)	*P*	OR	95%CI	N (%)	*P*	OR	95%CI
GLOBAL	AA	97 (24.15)	139 (35.01)	< 0.001	1.464	1.093–1.961	45 (33.83)	0.008	1.606	1.051–2.152	63 (35.80)	< 0.001	1.497	1.042–2.149
	AB	246 (61.12)	251 (63.22)	NS	1.035	0.828–1.292	87 (65.41)	NS	1.070	0.783–1.462	111 (63.07)	NS	1.032	0.776–1.371
	BB	59 (14.73)	7 (1.76)	< 0.001	0.120	0.055–0.264	1 (0.80)	< 0.001	0.051	0.007–0.371	2 (1.14)	< 0.001	0.077	0.018–0.310
WTHOUT OAPS	AA	97 (24.15)	106 (33.02)	0.040	1.367	1.003–1.863	39 (35.14)	0.008	1.701	1.086–2.666	46 (32.86)	0.040	1.360	0.913–2.026
	AB	246 (61.12)	211 (65.73)	NS	1.076	0.851–1.359	72 (64.86)	NS	1.061	0.759–1.484	96 (68.57)	NS	1.087	0.801–1.475
	BB	59 (14.73)	2 (1.25)	< 0.001	0.084	0.030–0.235	0 (0.00)	< 0.001	-	-	1 (0.71)	< 0.001	0.048	0.007–0.353

*KIR* killer cell immunoglobulin-like receptor, *RPL* recurrent pregnancy loss, *RIF* recurrent implantation failure, *uRF* unexplained recurrent fertility, *without OAPS* without fulfilling classification criteria for obstetric antiphospholipid syndrome.

After excluding patients with OAPS, a comparable distribution of KIR genotype was evident in the remaining cohort: KIR AB at 65.73% (211/321), KIR AA at 33.02% (106/321), and KIR BB at 1.25% (4/321).

### Centromeric KIR AA motif was the only Cen/Tel motif associated with the likelihood of uRF

The distribution of centromeric and telomeric motifs in women with uRF is outlined in Table 3. Importantly, in our cohort, cenAA was significantly increased with respect to the reference Spanish population (49.12% vs. 40.34%; p < 0.001). The cenAB was the most prevalent in the reference population.

**Table 3:** Centromeric and telomeric KIR haplotypes in the study groups and in the reference Spanish population.

	KIR centromeric and telomeric haplotypes frequencies in comparison with reference population												
	Ref. P. (n = 402)	uRF-group (n = 397)				RIF-group (n = 133)				RPL-group (n = 176)			
	N (%)	N (%)	*P*	OR	95%CI	N (%)	*P*	OR	95%CI	N (%)	*P*	OR	95%CI
cenAA	162 (40.34)	195 (49.12)	< 0.001	1.218	0,949–1,560	65 (48.87)	0.028	1.212	0.856–1.710	89 (50.57)	0.001	1.513	1.060–2.158
cenAB	178 (44.18)	171 (43.07)	NS	0.974	0.759–1.250	64 (48.12)	NS	1.089	0.770–1.537	71 (40.34)	NS	0.913	0.658–1.264
cenBB	42 (10.61)	31 (7.81)	NS	0.735	0.454–1.187	5 (3.01)	0.001	0.283	0.099–0.802	16 (9.09)	NS	0,855	0.460–1.556
telAA	211 (55.07)	227 (57.18)	NS	1.038	0.825–1.306	76 (57.14)	NS	1.037	0.750–1.436	102 (57.95)	NS	1.052	0.785–1.410
telAB	146 (36.23)	157 (39.55)	NS	1.084	0.833–1.411	54 (40.60)	NS	1.121	0.776–1.618	54 (30.68)	NS	0.847	0.592–1.211
telBB	15 (3.83)	14 (3.53)	NS	0.912	0.439–1.894	3 (2.26)	NS	0.584	0.167–2.034	6 (3.41)	NS	0.882	0.339–2.291

Ref. P. refers con control population data.

*OR* odds ratio, *CI* confidence interval, *RPL* recurrent pregnancy loss, *RIF* recurrent implantation failure, *uRF* unexplained recurrent fertility.

The presence of a cenAA motif was significantly associated with increased risk of being in the uRF-group (OR: 1.18; CI 0.949–1.560, *p* < 0.001). No significant differences in the frequencies of other centromeric and telomeric motifs were observed between patients in the uRF-group and the reference population.

Compared to the reference population, the frequency of the cenAA motif was significantly higher in the RIF-group (48.87% vs. 40.34%, *p* = 0.028) and the RPL-group (50.57% vs. 40.34%, *p* = 0.001) (Table 3 and Supplementary Table 1). The presence of a cenAA motif significantly increased the likelihood of being in the RIF (OR: 1.212; 0.856–1.710) and RPL (OR: 1.513; CI 1.060–2.158) groups.

Conversely, we observed that the frequency of the cenBB motif was significantly lower in the RIF-group (3.01% vs. 10.61%; *p* = 0.001) compared to the reference population, and this was consequently associated with a significantly reduced likelihood of being in the RIF group (OR: 0.283; CI 0.099–0.802).

The frequencies of the other cen/tel motifs among women in the S-, RPL- and FD-groups were similar to those described for the reference population (Table 3 and Supplementary Table 1).

### HLA-C distribution in couples with known fertility and uRF

We performed a comprehensive analysis of HLA-C haplotypes in both women and their male partners across all the studied groups, as outlined in Table 4, compared to our control group of fertile couples. The frequency of the C1 haplotype was significantly lower in women from the uRF-group (26.44% vs. 48.27%, *p* = 0.01) and the RPL-group (26.70% vs. 48.27%, *p* = 0.018). Similarly, the frequency of the C1 haplotype was significantly lower in the male partners within the uRF-group (22.92% vs. 62.06%, *p* < 0.001), as well as in both the RPL (22.72% vs. 62.06%, *p* < 0.001) and RIF (31.25% vs. 62.06%, *p* = 0.002) subgroups when compared to the control group. Conversely, there was a statistically significant increase of the C2 haplotype in the RIF-group (27.08% vs. 6.89%, *p* = 0.022) compared to the control group. In addition, we noticed an upward trend of the C2 haplotype in the partners of the uRF and RPL groups.

**Table 4:** Comparison of the frequency of the HLA-C1C1, C1C2 and C2C2 haplotypes between women groups (patients and controls) and men groups (patients and control partner).

HLA haplotype/Groups	Control	uRF-group				RPL-group				RIF-group			
	N (%)	N (%)	*P*	OR	CI 95%	N (%)	*P*	OR	CI 95%	N (%)	*P*	OR	CI 95%
C1C1 women	14 (48.27)	105 (26.44)	0.01	0.385	0.179–0.825	47 (26.70)	0.018	0.390	0.175–0.869	32 (33.33)	NS	0.535	0.230–1.244
C1C1 partner	18 (62.06)	91 (22.92)	< 0.001	0.181	0.082–0.398	40 (22.72)	< 0.001	0.179	0.078–0.411	30 (31.25)	0.002	0.277	0.116–0.660
C1C2 women	10 (34.48)	152 (38.04)	NS	1.178	0.533–2.602	68 (38.64)	NS	1.196	0.525–2.726	47 (48.95)	NS	1.822	0.768–4.324
C1C2 partner	9 (31.03)	159 (40.05)	NS	1.484	0.659–3.343	78 (44.32)	NS	1.768	0.762–4.101	40 (41.67)	NS	1.587	0.655–3.846
C2C2 women	5 (17.24)	54 (13.60)	NS	0.755	0.276–2.065	26 (14.77)	NS	0.832	0.291–2.376	17 (17.71)	NS	1.032	0.344–3.093
C2C2 partner	2 (6.89)	60 (15.11)	NS	0.240	0.126–0.455	23 (13.06)	NS	1.990	0.443–8.934	26 (27.08)	0.022	5.014	1.113–22.590

*OR* odds ratio, *CI* confidence interval, *RPL* recurrent pregnancy loss, *RIF* recurrent implantation failure, *uRF* unexplained recurrent fertility.

Interestingly, the risk of RPL and RIF was significantly lower in male partners who possessed the C1 haplotype (OR: 0.179, CI 0.078–0.411; OR: 0.277, CI 0.116–0.660, respectively).

### KIR and HLA-C combinations among couples with known fertility and uRF

We first focused on de combination HLA-C between women and partners (Supplementary Table 2). A significantly lower C1/C1 combinations was observed in the uRF-Group (9.03% vs. 27.59%, *p* = 0.004), RPL-Group (7.09% vs. 27.59%, *p* < 0.001), and RIF-Group (10.42% vs. 27.59%, *p* = 0.002) compared to the control group. Conversely, the frequency of HLA-C2/C2 partners was significantly higher in the RIF group, with a risk that was five times greater than that of the control group.

We then build a matrix of HLA-C haplotypes in the couples and maternal KIR across all the studied groups and compared to the fertile control group, as presented in Table 5 and Supplementary Table 3. We observed an increased frequency of at least one paternal C2 in the uRF-group (70.60% vs. 37.93%, *p* < 0.001), in RPL (68.75% vs. 37.93%, *p* = 0.003), and in RIF (71.63% vs. 37.93%, *p* < 0.001) groups compared with the control group. There was a significantly higher frequency of C2 haplotype in males of the RIF-group (27.08% vs. 6.89%, *p* = 0.022) compared to the control group, and an increasing trend in male partners of the uRF and RPL groups. Interestingly, this trend was not observed when considering only maternal C2.

**Table 5:** Matrix of combinations between KIR haplotypes and HLA-C1 and C2 in the uRF and in the control group.

KIR haplotype	HLA combination/Groups	C-group		uRF-group		
		N (%)	N (%)	*P*	OR	CI 95%
cenAA	C1C1/C1C1	5 (17.24)	11 (3.55)	< 0.001	0.176	0.056–0.549
	C1C1/C1C2	4 (13.79)	25 (8.06)	NS	0.548	0.176–1.700
	C1C1/C2C2	1 (3.45)	8 (2.58)	NS	0.741	0.089–6.146
	C1C2/C1C1	2 (6.90)	28 (9.03)	NS	1.340	0.302–5,935
	C1C2/C1C2	1 (3.45)	40 (12.90)	NS	4.148	0.549–31.337
	C1C2/C2C2	0 (0.00)	16 (5.16)	NS	–	–
	C2C2/C1C1	2 (6.89)	9 (2.90)	NS	0.837	0.102–6.850
	C2C2/C1C2	1 (3.45)	12 (3.87)	NS	0.543	0.115–2.556
	C2C2/C2C2	0 (0.00)	1 (0.32)	NS	–	–

		N (%)	N (%)	*P*	OR	CI 95%
cenAB	C1C1/C1C1	2 (6.89)	15 (4.84)	NS	0.686	0.149–3.161
	C1C1/C1C2	0 (0.00)	20 (6.45)	NS	–	–
	C1C1/C2C2	0 (0.00)	15 (4.84)	NS	–	–
	C1C2/C1C1	4 (13.79)	10 (3.23)	0.004	0.208	0.061–0.712
	C1C2/C1C2	1 (3.45)	31 (10.00)	NS	3.111	0.409–23.662
	C1C2/C2C2	0 (0,00)	14 (4.52)	NS	–	–
	C2C2/C1C1	1 (3.45)	9 (2.90)	NS	0.837	0.102–6.851
	C2C2/C1C2	0 (0.00)	14 (4.52)	NS	–	–
	C2C2/C2C2	0 (0.00)	4 (1.29)	NS	–	–

		N (%)	N (%)	*P*	OR	CI 95%
cenBB	C1C1/C1C1	1 (3.45)	2 (0.65)	NS	0.181	0.016–2.068
	C1C1/C1C2	1 (3.45)	8 (2.58)	NS	0.741	0.089–6,146
	C1C1/C2C2	0 (0.00)	1 (0.32)	NS	–	–
	C1C2/C1C1	1 (3.45)	5 (1.61)	NS	0.459	0.051–4.067
	C1C2/C1C2	1 (3.45)	6 (1.94)	NS	0.552	0.064–4.754
	C1C2/C2C2	0 (0.00)	1 (0.32)	NS	–	–
	C2C2/C1C1	0 (0.00)	2 (0.65)	NS	–	–
	C2C2/C1C2	0 (0.00)	3 (0.97)	NS	–	–
	C2C2/C2C2	1 (3.45)	0 (0.00)	NS	–	–

*OR* odds ratio, *CI* confidence interval, *uRF* unexplained recurrent fertility.

Conversely, the C1 haplotype was less prevalent in women from the uRF-group (26.44% vs. 48.27%, *p* = 0.01) and the RPL-group (26.70% vs. 48.27%, *p* = 0.018), as well as in their partners in the uRF-group (22.92% vs. 62.06%, *p* < 0.001), and both RPL (22.72% vs. 62.06%, *p* < 0.001) and RIF (31.25% vs. 62.06%, *p* = 0.002) subgroups when compared to the control group. Moreover, partners with the C1 haplotype had a significantly lower risk of RPL and RIF (OR: 0.179, CI 0.078–0.411; OR: 0.277, CI 0.116–0.660, respectively).

The cenBB motif was significantly more common in couples with known fertility compared to patients in the uRF-group (18.01% vs. 7.81%; *p* = 0.040) and the RIF-group (18.01% vs. 3.01%; *p* = 0.004). Although the cenBB motif was also more frequent in controls compared to patients in the RPL-group and FD-group, this difference did not reach statistical significance.

The frequency of the cenAA/C1C1 combination and C1C1 partners was significantly higher in the control group compared with uRPL (Table 5), with the RIF-Group and RPL-Group (33.3% vs. 9.01%; *p*: 0.048; and 33.3% vs. 6.85%; *p* = 0.009, respectively) (Supplementary Table 3). Women with the cenAA/C1C1 combination and HLA-C1C1 partners had almost a fivefold higher association with a successful reproductive outcome (OR: 0.220, CI 0.050–0.961) and (OR: 0.202, (CI 0.046–0.871), respectively).

## Discussion

In this comprehensive Spanish case–control study encompassing women experiencing uRF and their respective partners, we delved into the genetic patterns of KIR and the combinations of KIR and HLA-C alleles within the couples. Our findings validated previous data on the significantly elevated frequency of the KIR AA haplotype among women with uRF compared to a reference population outlined by Cisneros and Maftei in RIF^31,36^. Upon categorizing patients into four distinct, non-overlapping groups, we identified a heightened prevalence of the KIR AA haplotype in the S-Group, RPL-Group, and RIF-Group, coupled with an increased susceptibility to encountering any of these complications in women possessing the KIR AA haplotype. These results substantiate earlier studies^26,29,37^.

Moreover, we undertook a stratified analysis based on centromeric and telomeric zones. Despite the subdivision of the four groups into six additional combinations according to these motifs, cenAA emerged as a better biomarker for delineating the risk of RPL and RIF. Indeed, we demonstrated a significant increase of cenAA in the RIF, RLP, and S-Groups compared to the reference population.

This discovery may address the KIR Bx gap identified in the Morin study, as in our cohort, at least 56 patients with the cenAA haplotype but a classical AB haplotype (20 patients from the RIF-Group and 26 from the RPL-Group) might have been overlooked using the traditional nomenclature. This implies a potential loss of information concerning 14% of classic AB KIR patients. Consequently, our study emphasizes the significance of cen/tel classification, offering valuable clinical insights.

Furthermore, our research unveiled those women experiencing RPL and RIF demonstrated a diminished frequency of KIR BB, resulting in lower odds of encountering these complications. The KIR BB profile encompasses one or more activating receptors, and this shift in the KIR repertoire towards an activating phenotype may confer protective effects during early pregnancy. It is noteworthy that, unlike the KIR AA haplotype, which comprises AA genes in both centromeric and telomeric regions, KIR AB may carry a cenAA haplotype. This nuance might be overlooked when patients are categorized based on classical haplotypes. The centromeric motif assumes particular significance in this context, as KIR2DL1 is present in both cenA and all B haplotypes.

Within KIR cenA haplotypes, there exists a unique absence of other alleles to balance the inhibitory signal of the 2DL1 KIR, whereas cenB harbors weak inhibitory and activator alleles^16^. This distribution of alleles may elucidate the observed protective effect of the cenB genotype against uRF, while cenA increases the likelihood of pregnancy disorders. Interestingly, our study, for the first time, identified an association between the centromeric region of KIR genes and RIF and RPL. Our analysis revealed no differences in any telomeric haplotype, suggesting that the examination of centromeric regions may offer a more accurate means of defining the risk of RIF and RPL in our Spanish cohort. In contrast, Hiby et al. reported a protective role of telB in RPL patients instead of cenB haplotypes^22^.

Traditionally, published studies have predominantly focused on the analysis of individual KIR receptors in isolation. However, our study underscores the importance of a comprehensive examination of the entire KIR genotype, offering a more nuanced understanding of NK cell behavior. NK cells respond to a combination of activating and inhibiting receptors, and this holistic knowledge bears significant clinical implications.

Lastly, we scrutinized the KIR-HLA-C combinations matrix, as certain combinations have been linked to the risk of pregnancy complications^8,11,13,38^. Our observations indicated a significantly reduced proportion of the KIR AA-C1C1 combination and C1C1 partners in the RIF and RPL-groups compared to the control group.

These findings suggest that the HLA-C1 haplotype may exert a protective effect in KIR cenAA women, leading to an elevated proportion of C2 alleles in the RPL and RIF groups, particularly in paternal C2 alleles. This aligns with observed paternal C2 effects in other obstetrical morbidities such as eclampsia^22^. Indeed, the KIR receptors in haplotype A that bind to C1 alleles, namely 2DL2 and 2DL3, are weaker and less specific than 2DL1 with C2. Despite exploring additional KIR/HLA-C combinations, the differences did not reach statistical significance. The observation that HLA C1C1 increases in the control group when KIR AA is prevalent may reinforce the significance of this immunological code recognition in ensuring pregnancy success, presenting broader evolutionary implications.

In couples where the maternal KIR genotype is AA, the observed combination may signify a compensatory mechanism. In populations with a high prevalence of KIR AA, our results demonstrate a corresponding increase in the C1C1 combination. This suggests an intricate interplay between maternal KIR AA and the prevalence of C1C1.

In summary, a key finding of this study is that cenAA could represent a better biomarker of adverse pregnancy outcomes than classical AA, and might be particularly useful to identify women at increased risk among the heterogeneous KIR AB or Bx population. When the cenAA is expressed in women, the HLA-C1C1 combination is significantly more common in control women and in their partners, in contrast to cases of uRF, RPL, and RIF. This co-presence acts as a protective factor, as it does not serve as the specific ligand for the main inhibitory receptor of the cenAA (2DL1). Consequently, this receptor does not exert its potentially detrimental function in NK cells.

### Supplementary Information

Supplementary Information.

## Data Availability

The datasets generated and analyzed during the current study are available from the corresponding author on reasonable request.
