# Supplementary material for: Centromeric AA motif in KIR as an optimal surrogate marker for precision definition of alloimmune reproductive failure
Source: Sci Rep. 2024 Feb 9;14:3354. doi: 10.1038/s41598-024-53766-x (PMC10858137; doi:10.1038/s41598-024-53766-x)
Supplement: Supplementary file 1 — Supplementary Information. [file 41598_2024_53766_MOESM1_ESM.docx]

| **KIR CENTROMERIC AND TELOMERIC HAPLOTYPES FREQUENCIES IN COMPARISON WITH REFERENCE POPULATION** | | | | | | | | | |
| --- | --- | --- | --- | --- | --- | --- | --- | --- | --- |
|  | **Ref. P. (n=402)** | **S-group (n=60)** | | | | **FD-group (n=28)** | | | |
|  |  |  |  |  |  |  |  |  |  |
|  | **N (%)** | **N (%)** | **P** | **OR** | **95%CI** | **N (%)** | **P** | **OR** | **95%CI** |
|  |  |  |  |  |  |  |  |  |  |
| **cenAA** | 162 (40.34) | 29 (48.33) | NS | 1.198 | 0.742-1.933 | 12 (42.86) | NS | 1.062 | 0.614-2.939 |
|  |  |  |  |  |  |  |  |  |  |
| **cenAB** | 178 (44.18) | 22 (36.67) | NS | 0.830 | 0.493-1.393 | 14 (50.00) | NS | 1.110 | 0.607-2.029 |
|  |  |  |  |  |  |  |  |  |  |
| **cenBB** | 42 (10.61) | 9 (15.00) | NS | 1.411 | 0.655-3.037 | 2 (7.14) | NS | - | 0-0.802 |
|  |  |  |  |  |  |  |  |  |  |
| **telAA** | 211 (55.07) | 33 (55.00) | NS | 0.999 | 0.634-1.573 | 16 (57.14) | NS | 0.490 | 0.550-1.958 |
|  |  |  |  |  |  |  |  |  |  |
| **telAB** | 146 (36.23) | 23 (38.33) | NS | 1.058 | 0.632-1.772 | 11 (39.29) | NS | 1.084 | 0.527-2.232 |
|  |  |  |  |  |  |  |  |  |  |
| **telBB** | 15 (3.83) | 4 (6.67) | NS | 1.725 | 0.558-5.332 | 1 (3.57) | NS | 0.924 | 0.118-7.223 |

**Supplementary Table 1.** Cen/tel haplotype in sterility- and fetal death-groups.

Ref. P.: control population data; OR, odds ratio; CI, confidence interval. RPL: recurrent pregnancy loss; S-group: sterility group; FD-group: fetal death group.

**Supplementary Table 2.** Combination of HLA-C1 and HLA-C2 in the study groups and in the control group.

| **HLA**  **combination/ Groups** | **C-group** |  | **uRF-group** | |  |  | **RPL-group** | |  |  | **RIF-group** | |  |
| --- | --- | --- | --- | --- | --- | --- | --- | --- | --- | --- | --- | --- | --- |
|  | **N (%)** | **N (%)** | **P** | **OR** | **95% CI** | **N (%)** | **P** | **OR** | **95% CI** | **N(%)** | **P** | **OR** | **95% CI** |
| **C1C1/C1C1** | 8 (27.59) | 28 (9.03) | 0.004 | 0.260 | 0.105-0.642 | 10 (7.09) | <0,001 | 0.200 | 0.071-0.565 | 10 (10.42) | 0.002 | 0.305 | 0.107-0.867 |
| **C1C1/C1C2** | 5 (17.24) | 52 (16.77) | NS | 0.927 | 0.337-2.550 | 24 (17.02) | NS | 0.943 | 0.326-2.729 | 13 (13.54) | NS | 0.751 | 0.243-2.320 |
| **C1C1/C2C2** | 1 (3.45) | 24 (7.74) | NS | 2.349 | 0.306-18.029 | 13 (9.22) | NS | 2.843 | 0.357-22.642 | 9 (9.38) | NS | 2.896 | 0.351-23.878 |
| **C1C2/C1C1** | 7 (24.14) | 43 (13.87) | NS | 0.506 | 0.203-1.256 | 23 (16.31) | NS | 0.612 | 0.234-1.601 | 11 (11.46) | NS | 0.406 | 0.141-1.170 |
| **C1C2/C1C2** | 3 (10.34) | 77 (24.84) | NS | 2.864 | 0.843-9.726 | 38 (26.95) | 0.050 | 3.197 | 0.914-11.178 | 20 (20.83) | NS | 2.280 | 0.626-8.307 |
| **C1C2/C2C2** | 0 (0.00) | 31 (10.00) | NS | - | - | 7 (4.96) | NS | - | - | 16 (16.67) | 0.019 | - | - |
| **C2C2/C1C1** | 3 (10.34) | 20 (6.40) | NS | 0.597 | 0.166-2.145 | 7 (4.96) | NS | 0.452 | 0.109-1.866 | 9 (9.38) | NS | 0.896 | 0.226-3.557 |
| **C2C2/C1C2** | 1 (3.45) | 30 (9.68) | NS | 3.000 | 0.394-22.840 | 9 (11.35) | NS | 1.909 | 0.232-15.682 | 7 (7.29) | NS | 2.202 | 0.259-18.679 |
| **C2C2/C2C2** | 1 (3.45) | 5 (1.61) | NS | 0.459 | 0.051-4.067 | 3 (2.13) | NS | 0.608 | 0.061-6.067 | 1 (1.04) | NS | 0.294 | 0.017-4.864 |

OR, odds ratio; CI, confidence interval. RPL: recurrent pregnancy loss; RIF: recurrent implantation failure; uRF: unexplained recurrent fertility

**Supplementary Table 3.** Matrix of combinations between KIR haplotypes and HLA-C1 and C2 in the study groups and in the control group.

|  | **HLA** |  |  |  |  |  |  |  |  |  |  |  |  |  |  |
| --- | --- | --- | --- | --- | --- | --- | --- | --- | --- | --- | --- | --- | --- | --- | --- |
| **KIR** | **combination/ Groups** | **C-group** | **uRF-group** |  |  |  | **RPL-group** |  |  |  | **RIF-group** |  |  |  |  |
| **haplotype** |  |  |  |  |  |  |  |  |  |  |  |  |  |  |  |
|  |  | **N (%)** | **N (%)** | **P** | **OR** | **CI 95%** | **N (%)** | **P** | **OR** | **CI 95%** | **N (%)** | **P** | **OR** | **CI 95%** |  |
|  | **C1C1/C1C1** | 5 (17.24) | 11 (3.55) | <0.001 | 0.176 | 0.056-0.549 | 5 (3.54) | 0.004 | 0.176 | 0.047-0.656 | 4 (4.17) | 0.015 | 0.208 | 0.052-0.837 |  |
|  | **C1C1/C1C2** | 4 (13.79) | 25 (8.06) | NS | 0.548 | 0.176-1.700 | 12 (8.51) | NS | 0.581 | 0.173-1.949 | 5 (5.21) | NS | 0.343 | 0.085-1.375 |  |
|  | **C1C1/C2C2** | 1 (3.45) | 8 (2.58) | NS | 0.741 | 0.089-6.146 | 4 (2.84) | NS | 0.817 | 0.088-7.593 | 4 (4.17) | NS | 1.217 | 0.130-11.342 |  |
|  | **C1C2/C1C1** | 2 (6.90) | 28 (9.03) | NS | 1.340 | 0.302-5,935 | 16 (11.34) | NS | 1.728 | 0.375-7.962 | 9 (9.38) | NS | 1.396 | 0.284-6.861 |  |
|  | **C1C2/C1C2** | 1 (3.45) | 40 (12.90) | NS | 4.148 | 0.549-31.337 | 21 (14.89) | NS | 4.900 | 0.632-37.981 | 10 (10.42) | NS | 3.255 | 0.398-26.572 |  |
|  | **C1C2/C2C2** | 0 (0.00) | 16 (5.16) | NS | - | - | 4 (2.84) | NS | - | - | 6 (6.25) | NS | - | - |  |
|  | **C2C2/C1C1** | 2 (6.89) | 9 (2.90) | NS | 0.837 | 0.102-6.850 | 3 (2.13) | NS | 0.293 | 0.046-1.840 | 4 (4.17) | NS | 0.587 | 0.101-3.380 |  |
|  | **C2C2/C1C2** | 1 (3.45) | 12 (3.87) | NS | 0.543 | 0.115-2.556 | 7 (4.96) | NS | 1.462 | 0.173-12.364 | 2 (2.08) | NS | 0.595 | 0.052-6.816 |  |
| **cenAA** | **C2C2/C2C2** | 0 (0.00) | 1 (0.32) | NS | - | - | 1 (0.71) | NS | - | - | 0 (0.00) | NS | - | - |  |
|  |  | **N (%)** | **N(%)** | **P** | **OR** | **CI 95%** | **N(%)** | **P** | **OR** | **CI 95%** | **N(%)** | **P** | **OR** | **CI 95%** |  |
|  |  |  |  |  |  |  |  |  |  |  |  |  |  |  |  |
|  | **C1C1/C1C1** | 2 (6.89) | 15 (4.84) | NS | 0.686 | 0.149-3.161 | 4 (2.84) | NS | 0.394 | 0.068-2.261 | 6 (6.25) | NS | 0.900 | 0.171-4.719 |  |
|  | **C1C1/C1C2** | 0 (0.00) | 20 (6.45) | NS | - | - | 7 (4.96) | NS | - | - | 8 (8.33) | NS | - | - |  |
|  | **C1C1/C2C2** | 0 (0.00) | 15 (4.84) | NS | - | - | 8 (5.67) | NS | - | - | 5 (5.21) | NS | - | - |  |
|  | **C1C2/C1C1** | 4 (13.79) | 10 (3.23) | 0.004 | 0.208 | 0.061-0.712 | 3 (2.13) | 0.004 | 0.135 | 0.028-0.644 | 2 (2.08) | 0.009 | 0.133 | 0.023-0.768 |  |
| **cenAB** | **C1C2/C1C2** | 1 (3.45) | 31 (10.00) | NS | 3.111 | 0.409-23.662 | 15 (10.64) | NS | 3.333 | 0.422-26.291 | 8 (8.33) | NS | 2.545 | 0.304-21.247 |  |
|  | **C1C2/C2C2** | 0 (0,00) | 14 (4.52) | NS | - | - | 3 (2.13) | NS | - | - | 9 (9.38) | NS | - | - |  |
|  | **C2C2/C1C1** | 1 (3.45) | 9 (2.90) | NS | 0.837 | 0.102-6.851 | 3 (2.13) | NS | 0.608 | 0.061-6.067 | 5 (5.21) | NS | 1.538 | 0.172-13.725 |  |
|  | **C2C2/C1C2** | 0 (0.00) | 14 (4.52) | NS | - | - | 7 (4.96) | NS | - | - | 5 (5.21) | NS | - | - |  |
|  | **C2C2/C2C2** | 0 (0.00) | 4 (1.29) | NS | - | - | 2 (1.42) | NS | - | - | 1 (1.04) | NS | - | - |  |
|  |  | **N (%)** | **N (%)** | **P** | **OR** | **CI 95%** | **N (%)** | **P** | **OR** | **CI 95%** | **N (%)** | **P** | **OR** | **CI 95%** |  |
|  |  |  |  |  |  |  |  |  |  |  |  |  |  |  |  |
|  | **C1C1/C1C1** | 1 (3.45) | 2 (0.65) | NS | 0.181 | 0.016-2.068 | 1 (0.71) | NS | 0.200 | 0.012-3.293 | 0 (0.00) | NS | - | - |  |
|  | **C1C1/C1C2** | 1 (3.45) | 8 (2.58) | NS | 0.741 | 0.089-6,146 | 5 (3.54) | NS | 1.029 | 0.115-9.154 | 0 (0.00) | NS | - | - |  |
|  | **C1C1/C2C2** | 0 (0.00) | 1 (0.32) | NS | - | - | 1 (0.71) | NS | - | - | 0 (0.00) | NS | - | - |  |
|  | **C1C2/C1C1** | 1 (3.45) | 5 (1.61) | NS | 0.459 | 0.051-4.067 | 4 (2.84) | NS | 0.817 | 0.088-7.593 | 0 (0.00) | NS | - | - |  |
|  | **C1C2/C1C2** | 1 (3.45) | 6 (1.94) | NS | 0.552 | 0.064-4.754 | 2 (1.42) | NS | 0.402 | 0.035-4.597 | 2 (2.08) | NS | 0.595 | 0.052-6.816 |  |
|  | **C1C2/C2C2** | 0 (0.00) | 1 (0.32) | NS | - | - | 0 (0.00) | NS | - | - | 1 (1.04) | NS | - | - |  |
|  | **C2C2/C1C1** | 0 (0.00) | 2 (0.65) | NS | - | - | 1 (0.71) | NS | - | - | 0 (0.00) | NS | - | - |  |
| **cenBB** | **C2C2/C1C2** | 0 (0.00) | 3 (0.97) | NS | - | - | 2 (1.42) | NS | - | - | 0 (0.00) | NS | - | - |  |
|  | **C2C2/C2C2** | 1 (3.45) | 0 (0.00) | NS | - | - | 0 (0.00) | NS | - | - | 0 (0.00) | NS | - | - |  |

OR, odds ratio; CI, confidence interval. RPL: recurrent pregnancy loss; RIF: recurrent implantation failure; uRF: unexplained recurrent fertility
